# Monoclonal Antibodies and Immunoassay for Medical Plant-Derived Natural Products: A Review

**DOI:** 10.3390/molecules22030355

**Published:** 2017-02-26

**Authors:** Xin Yan, Yan Zhao, Yue Zhang, Huihua Qu

**Affiliations:** 1 School of Chinese Materia Medica, Beijing University of Chinese Medicine, Beijing 100029, China; 20150931805@bucm.edu.cn (X.Y.); jinzy0423@163.com (Y.Z.); 2 School of Basic Medical Sciences, Beijing University of Chinese Medicine, Beijing 100029, China; zhaoyandr@gmail.com; 3 Center of Scientific Experiment, Beijing University of Chinese Medicine, Beijing 100029, China

**Keywords:** medical plant-derived natural products, monoclonal antibody, immunoassay

## Abstract

Owing to the widespread application value, monoclonal antibodies (MAbs) have become a tool of increasing importance in modern bioscience research since their emergence. Recently, some researchers have focused on the production of MAbs against medical plant-derived natural products (MPNP), the secondary metabolites of medical plants. At the same time, various immunoassay methods were established on the basis of these MPNP MAbs, and then rapidly developed into a novel technique for medical plant and phytomedicine research in the area of quality control, pharmacological analysis, drug discovery, and so on. Dependent on the research works carried out in recent years, this paper aims to provide a comprehensive review of MAbs against MPNP and the application of various immunoassay methods established on the basis of these MAbs, and conclude with a short section on future prospects and research trends in this area.

## 1. Introduction

Within clinical medication practice, whether in traditional Chinese medicine (TCM), Japanese Kampo medicine, or Korean medicine, medical plants and phytomedicines are the most important aspect in terms of both type and usage amount, and also possesses a long history of safe and efficacious administration worldwide. It has been reported that 14% of people around the world use phytomedicines and this level is growing continuously [1]. 

As the secondary metabolites of medical plants, medical plant-derived natural products (MPNP) play key roles in multiple areas of research, ranging from investigating the biologically active principles and quality control of phytomedicines, to pharmacodynamics studies, metabolic processes of drugs, and drug discovery [2]. Notable examples include paclitaxel and its derivatives from yew (*Taxus*) species [3], and the important antimalarial and potential anti-cancer agent artemisinin, originally derived from the traditional Chinese herb *Artemisia annua* L. [4], has clearly demonstrated the importance of MPNP. With the increased employ, requirement, and study of MPNP came greater requests for convenient, efficient, and sensitive analysis and detection technologies. However, the reality is that although the past few decades have seen major advances and tremendous achievements of conventional analysis techniques, represented by High Performance Liquid Chromatography (HPLC), there are still some shortcomings, such as complex pretreatment, time consumption, and high requirements with respect to instruments that are unable to satisfy those requirements above. 

Monoclonal antibodies (MAbs) are monovalent antibodies which bind to the same epitope and are produced from a single B-lymphocyte clone. In 1975, professors Köhler and Milstein [5] successfully prepared anti-sheep red cell monoclonal antibodies for the first time, and then set up a method for preparing monoclonal antibodies by the hybridoma technique. MAbs are often considered superior to polyclonal antibodies because of their specificity to a single epitope, their homogeneous structure, and their ability to be mass-produced. The advent of monoclonal antibody technology not only brings a revolution in the field of immunology in the biomedical sciences, but also promotes the development of many disciplines. Antibody-based bioanalytical measurement and separation techniques have been routinely used in medical and clinical settings, and the targets to which immune analytical tools are being applied have covered a wide spectrum of compounds such as proteins, pesticides, biomarkers, and heavy metals, and are expanding continuously [6].

Recently, with the rapid development of the molecular biosciences and their biotechnological applications, MAbs against MPNP have been produced continuously. At the same time, immunoassay methods, such as ELISA, immunoaffinity chromatography, and chromatographic immunostaining developed the basis upon which these anti-MPNP MAbs have become an important analytical tool, because of their specificity, for quality control, pharmacokinetics research, and quantitative and qualitative analysis of phytomedicine. Additionally, as new materials, such as colloidal gold, optical phosphors, and quantum dots (QDs), become available, immunoassays and biosensors are quick to adopt these new technologies and, as a consequence, further improve the detection of MPNP in terms of accuracy, sensitivity, and convenience. 

In 1993, an anti-taxol antibody was generated, which was considered to be the first MAbs for MPNP [7]. Since then, the production of anti-MPNP MAbs has seen quite significant development. The broad applicability of these MAbs was investigated continuously. This report reviews, for the first time, the recent advances in the field of MAbs against MPNP, and also selects several examples to illustrate the established immunoassay methods on the basis of these produced anti-MPNP MAbs with promising applications as analytical tools and superior alternatives to existing conventional analytical strategies. The paper concludes by presenting future prospects in this field.

## 2. Synthesis of Artificial Antigens

Theoretically, a small molecule (<1000 Da) cannot elicit immune responses. Most of the MPNP are poorly immunogenic because they are low molecular weight compounds, a so-called hapten. Therefore, the synthesis of artificial antigens by coupling with carrier macromolecules, such as proteins, leads them to be recognized and be phagocytosed by antigen-presenting cells, which is the committed step during the production of MAbs. It is important that the immunizing hapten selected or designed should preserve the common structure of MPNP as much as possible to obtain a high specificity with high affinity antibodies.

### 2.1. Coupling Method between Carrier Protein and Hapten

During the synthesis of MPNP artificial antigens, appropriate functional groups (i.e., carboxyl acid, amino, hydroxyl, sulfhydryl), which could be inherent or introduced, were used as a side arm for covalent binding with the carrier protein using various reaction schemes. Including periodate oxidation, the carbodiimide method, mixed anhydrides reaction, glutaraldehyde reaction, and the succinate method, several methods have been commonly used for the coupling between carrier proteins and MPNP (Table 1).

The selection of an appropriate coupling method that is suitable for the individual structural characteristics of different MPNP is crucially important for the synthesis of artificial antigens, and will significantly influence the specificity of MAbs. For example, using the periodate oxidation conjugate method, a MAbs against daidzin (DA) displayed a cross-reactivity profile with DA-related compounds, including daidzein (16.16%), genistin (82.35%), genistein (24.42%), glycitin (11.92%), and puerarin (3.37%) [24]. In contrast, taking advantage of Mannich condensation, Yusakul et al. [37] obtained a highly-specific anti-DA MAbs showing lower cross-reactivity with daidzein (1.57%) and glycitin (0.48%) compared to the former.

#### 2.1.1. Sodium Periodate Oxidation Method

Sodium periodate oxidation reaction is the most widely used coupling method in the artificial antigen synthesis of MPNP. Sugar moieties could rapidly convert to aldehydes via periodate oxidation and then conjugate with the lysine or arginine residues of bovine serum albumin (BSA) under alkaline conditions [41]. Among MPNP, glycosides account for a large percentage. Due to the presence of the sugar part, these compounds are suitable for forming a chemical bond to carrier proteins through the sodium periodate oxidation reaction. 

For example, due to the possession of sugar moieties, the antigen of ginsenoside Re [8], glycyrrhizic acid [11], puerarin [12], and many other MPNP were obtained by such method.

#### 2.1.2. Carbodiimide Method

The carbodiimide method was commonly used to link the carboxyl groups of small molecular compounds to the amine groups of protein. For example, the carboxyl group in aristolochic acid-II reacted with the free amino group mainly of the lysine in BSA resulting in an aristolochic acid-II-BSA conjugate [29]. Such a method was also used for baicalin [27], chenodeoxycholic acid [26], and coptisine [31].

#### 2.1.3. Mixed Anhydrides Reaction (MAR)

MPNPs, like hyodeoxycholic acid [36], contain carboxylic acid groups that can react with isobutyl chloroformate to form mixed anhydride—an active intermediate. The amine functional groups of proteins are stable to acylation with anhydride reagents, eventually forming amide bonds [42]. 

#### 2.1.4. Active Ester Method (AEM)

Dicyclohexyl carbodiimide can be used to prepare active esters of carboxylate-containing compounds using *N*-hydroxysuccinimide (NHS). The reaction outcome is able to form amide linkages with carrier proteins [42]. Taking advantage of this method, an antigen of tetrahydrocannabinolic acid was synthesized successfully [43]. 

#### 2.1.5. Succinate Method

Succinylated derivatives of MPNP can be prepared by reaction of the succinic anhydride with available –OH groups. Possessing a carboxy group, such MPNP derivatives can conjugate with proteins as described above [42]. Using the succinate method, Kido et al. synthesized a succinate of aconitine possessing a carboxyl group, and conjugated with BSA successfully to produce an antigen [39]. 

#### 2.1.6. Mannich Condensation

Aldehydes may participate in a condensation reaction between a protein containing an amine and an MPNP containing a sufficiently-active hydrogen, yielding an alkylated derivative that effectively crosslinks the two molecules through the carbonyl group of the aldehyde [42]. The antigen of DA was produced successfully as described [44]. 

### 2.2. Identification of Artificial Antigen

In order to confirm whether the conjugate was synthesized successfully, and find out if there is a sufficient number of haptens and immunogenicity to raise specific antibodies, the identification of MPNP artificial antigen before immunization is indispensable.

#### 2.2.1. Matrix-Assisted Laser Desorption Ionization Time-of-Flight Mass Spectrometry (MALDI-TOF-MS)

**Figure 1:**
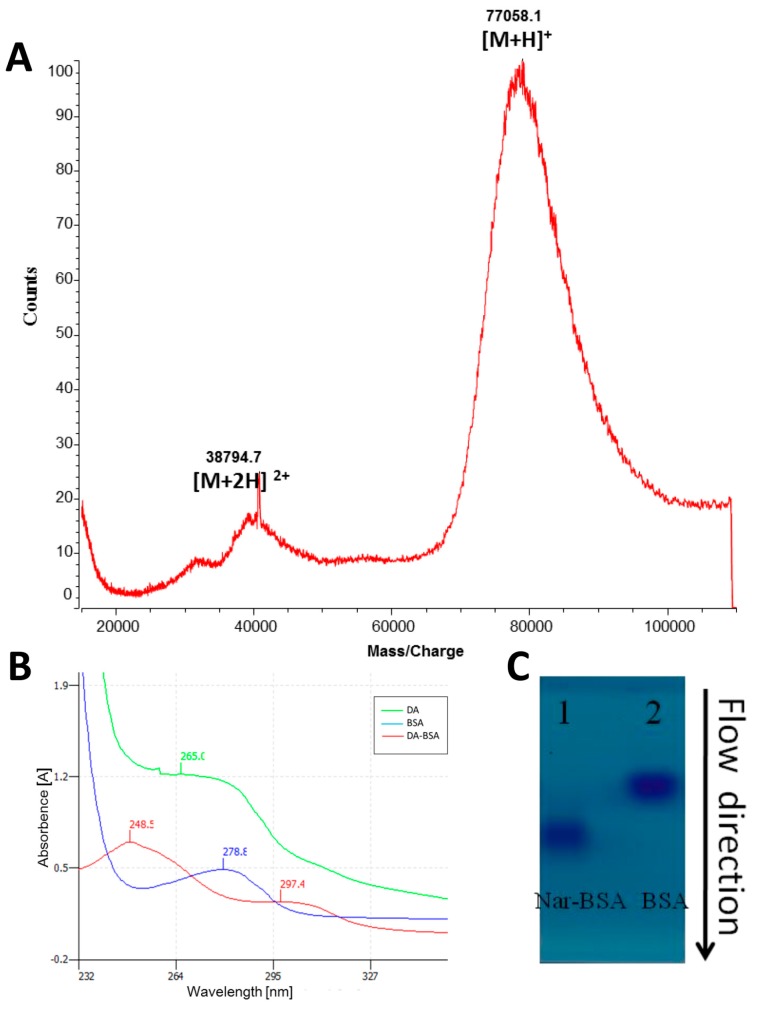
(**A**) Direct determination of the hapten number in the naringin-bovine serum albumin (Nar-BSA) conjugate by matrix-assisted laser desorption/ionization TOF mass spectrometry. [M + H]^+^ and [M + 2H]^2+^ are single- and double-protonated molecules of Nar-BSA, respectively [14]. Reprinted with permission from Huihua Qu et al. (2016), Copyright 2016 JSSC; (**B**) UV Spectrum of daidzin (DA), BSA, and DA-BSA [45]. Reprinted with permission from Huihua Qu et al. (2016), Copyright 2016 JSSC; (**C**) Analysis of Nar-BSA conjugate and carrier protein BSA via non-denaturing agarose electrophoresis. Lane 1: Nar-BSA conjugate, Lane 2: BSA [14].

MALDI-TOF-MS is an effective method routinely and widely employed by researchers now for the verification of the conjugates and the determination of the hapten number in a conjugate (Figure 1A). This strategy could be applied to the production of MAbs for MPNP, especially for those compounds having no specific UV absorbance, since difficulties and ambiguities remain in the confirmation of bio-conjugate formation. 

The conjugate of coptisine was determined by MALDI-TOF MS in a study [31]. Using experimental results and a molecular weight of 66,433 for BSA, the calculated value of the hapten component (MW 380) was *m*/*z* 1022, indicating at least three molecules of hapten conjugated with BSA. This hapten number was estimated to be enough for immunization.

#### 2.2.2. Ultraviolet Spectrum (UV) Analyses

During the production of MAbs against chenodeoxycholic acid (CDCA), UV analyses were used to identify the synthesized antigens. They revealed that the maximum absorption of BSA samples was located at 278 nm, and that CDCA exhibited almost no ultraviolet absorption. While the peak absorption for the CDCA-BSA was located at 252 nm, and the shape of the CDCA-BSA curve exhibited the curve characteristics of both CDCA and BSA, these results indicated that CDCA was conjugated with the BSA successfully [26]. 

In another study, full-wave UV spectrograms of the antigen conjugates of DA, BSA, and DA-BSA were detected (Figure 1B). The peak absorption for DA-BSA was 297 nm, different from DA (248 nm) and BSA (278 nm), which indicated the likely presence of a new substance and consequently successful synthesis [45].

#### 2.2.3. Agarose Gel Electrophoresis

Non-denaturing agarose gel electrophoresis was used to demonstrate that when as few as two molecules of an MPNP are attached to the carrier, the conjugate band migrates differently from that of the carrier alone or of the coupling reagent-treated carrier.

During the generation of an antigen for naringin (Nar), the conjugates were identified by non-denaturing agarose gel electrophoresis (Figure 1C). The migration velocity of the Nar-BSA conjugates was faster than that of the carrier protein (BSA) alone, which indicate that the Nar sample was successfully coupled to the carrier protein BSA [14]. 

## 3. Production of Anti-MPNP MAbs

Various methods, including conventional hybridoma technology and display technology (such as phage display, mammalian cell display, and polysome display) have been developed for the production of MAbs [46,47]. The most common technology adopted in the area of anti-MPNP MAbs production was the hybridoma technique, including the PEG and electrical fusion method. 

So far, the production of MAbs has been extended to a variety MPNPs, in which several categories including alkaloids, terpenes, quinones, bile acids, phenolic acids, and iridoids have been covered (Table 2). Notably, such as ginsenoside [8], glycyrrhizic acid [11] and puerarin [12], specific MAbs against some valuable compounds have been produced successfully. 

## 4. Specificity and Cross-Reactivity

Cross-reactivity is the most important factor in determining the value of an antibody and dominates the specificity of an immunoassay (Table 3). The specificity of the immunochemical method is largely determined by the specificity of the MAbs employed. Actually, lower cross-reactivity for chemically similar analogues means higher selectivity for the determination of a target compound. For example, the cross-reactivities of the anti-glycyrrhizic acid (GA) MAbs against glycyrrhetic acid-3-*O*-glucuronide and glycyrrhetic acid were 0.585% and 1.865%. The other three analogues were all less than 0.005%, respectively. This implies that this anti-GA-MAbs had a weak cross-reaction with those related compounds, but specifically reacted with GA [49]. Meanwhile, if the MAbs possess wide cross-reactivities against some other compounds, the immunoassay system established subsequently can only approximately reflect the total amount of target compounds even though it is much more sensitive than TLC and HPLC analysis.

### 4.1. Advantages of Cross-Reactivity

However, on the other hand, the cross-reactivity of MPNP-MAbs against related compounds also may become the special advantage of the antibody reagent used in the immunoassay, which could be applied to pharmacological study, synthesis of antigen conjugates, and so on.

#### 4.1.1. Use in Pharmacological Study

The MAbs against crocin produced by Xuan et al. showed wide cross-reactivities against crocetin glycosides [17]. However, this wide reactivity become the main advantage of the antibody used in ELISA, since it is better than a special antibody for the metabolic study of crocin and the study of pharmacologically-active mechanisms of crocin on long-term potentiation in the central nervous system.

In another report, anti-paeoniflorin MAbs showed higher reactivity to paeoniflorin and albiflorin, which are major constituents in peony root, than to those of oxypaeoniflorin and benzoylpaeoniflorin, which are the minor constituents, indicating that it is possible to apply quality control and standardization of pharmacological activity of a crude drug and its prescription using ELISA [51]. 

#### 4.1.2. Use in the Synthesis of Antigen Conjugate

Immunization of some MPNPs may be quite difficult in the common manner because they are not only of low molecular weight, but also have no applicable reactive group in the molecule. Under this condition, we can use some related compound which has a similar skeleton with the target compound as a hapten for the synthesis of antigen, and the MAbs produced then may cross-react with the target compound which means it can be used as a MAbs specific for the target compound. 

For example, a heterogeneous hapten, 9-*O*-carboxymethyl-berberrubine, which shares a part of the coptisine molecule, was designed as an intermediate for conjugation with a carrier protein. The MAbs produced later was applied in the ELISA method for determining the levels of coptisine in biological samples due to the high cross-reactivity, and the sensitivity and selectivity of this ELISA are confirmed to be sufficient [31]. Artemisinin has no suitable functional group as well. Artesunate, which is a structurally-related compound of artemisinin, was selected for an ideal hapten to prepare a conjugated protein. MAbs possessing cross-reactivity with artemisinin was obtained subsequently [30]. 

#### 4.1.3. Others Advantages

DA-MAbs prepared by Sakamotohas showed high cross-reactivity against genistin. Considering that DA and genistin are two of the major isoflavone glycosides in soybeans, they developed an ELISA based on the produced DA-MAbs which could be used for the determination of soy isoflavones contained in soy products thereby providing an approach to avoid overconsumption of soy isoflavones, which leads to the safe dietary intake of soy products [24]. 

In another case, the investigation of MAbs against delta-1-tetrahydrocannabinolic acid, two major metabolites of cannabinoids 7-0X0-A6-THC and 7-hydroxyl-A6-THC, had clear reactivities (393% and 69%, respectively) for the ELISA. Thus, this newly-established ELISA has a prospective application that might be applied for the first screening for urine samples of marihuana users [40]. 

## 5. Immunoassay for MPNP Using MAbs

Since 1993, various immunoassay systems using monoclonal antibodies have been established and have become important methodologies for studies of MPNP due to their multiple benefits, including inexpensiveness, speed, high specificity, and sensitivity over conventional analysis methods, such as HPLC, TLC, etc.

### 5.1. Enzyme-Linked Immunosorbent Assay (ELISA)

Although the conventional chromatographic methodologies have been proved to be accurate and sufficiently sensitive, there are some disadvantages, such as the limited number of samples, complex pretreatment, time, and labor consumption, besides interference by other compounds included in herbal medicines. In contrast, an ELISA using MAbs is faster, more convenient, and more economic compared with other methods. Despite their high cost and the long process involved in their development and establishment, ELISA has become an important methodology for the quantitative and/or qualitative analysis of MPNP having small molecular weights.

#### 5.1.1. Detection and Quality Control of Toxic Components

Medication safety is becoming an important issue during the application of herbal medicine [55,56,57]. With the objectives of ensuring the safe use of herbal medicines and reducing the side effects of toxic components as much as possible, there is a continuing and pressing need for economical analytical methods for the detection and quality control of toxic components, even in trace levels, in various herbal medication complex environmental and biological media. 

##### Aristolochic Acids

As is the major components of *Aristolochia* and *Asarum* species, aristolochic acids (AAs) have already been proven to possess nephrotoxicity [58] and carcinogenicity [59]. Shang et al. analyzed AA-II concentration in crude drugs derived from *Aristolochia* plants using MAbs against AA-II, and established an ELISA method for the quality control of crude drugs derived from *Aristolochia* plants [60]. 

##### Aconitine

Since the pharmacological activities and toxicities of Aconiti radixes depend on the total concentration of aconitine-type alkaloids, Kidoa et al. prepared a typical MAbs against aconitine that recognizes aconitine and aconitine-type alkaloids, and established an ELISA method using the new MAbs, which is convenient for quality control and appropriate use of Aconiti radixes.

##### Ginkgolic Acids

Ginkgolic acids, which could induce allergic contact dermatitis, is contained in the fruits and leaves of *Ginkgo biloba* Linn. Since Ginkgo leaf extracts are available commercially as a drug, the quality control for ginkgolic acids is necessary. Considering the characteristic of low concentration, an ELISA method for the quantitative determination of total ginkgolic acids content in ginkgo crude drugs with no interference from the sample matrix was developed using MAbs [10]. 

#### 5.1.2. Content Determination in Biological Samples or Drugs

Morinaga et al. established a new approach for one-step analysis of total sennosides (the major purgative constituents of rhubarb) concentration in rhubarb and senna samples by using the combination of anti-sennside A and anti-sennside B MAbs in a single ELISA [61]. In another investigation, the anti-paeoniflorin-MAbs was applied to determine the total concentration of paeoniflorin and albiflorin in various Chinese traditional medicines [51]. The application of the competitive ELISA using MAbs against tetrahydrocannabinolic acid for the judgment of marijuana sample detection of marijuana and distinguishing *Cannabis sativa* samples from different plant species was also reported [62]. 

#### 5.1.3. Harvesting and Breeding of Medicinal Plants

Sritularak et al. used the anti-ginsenoside Rb1(G-Rb1), anti-G-Rg1, and anti-G-Re MAbs to determine the amount of G-Rb1, G-Rg1, and G-Re in American ginseng berry and flower samples, which were harvested in various months throughout the year (American ginseng were collected from May to September 2005) to determine the best season for harvesting [63]. 

To confirm the homogeneity of GA concentrations in cultivated plants, Fujii et al. monitored GA concentrations over two years in seven plants containing 4.0 dw % of GA using the established ELISA, and suggested that the highest concentration of GA was 5.36 dw % [64]. 

#### 5.1.4. Metabolic Study

Huihua et al. developed a quick, specific, and sensitive ELISA to determine puerarin in human saliva by adding anti-puerarin MAbs to the reaction, and further applied this ELISA to the pharmacokinetic analysis of puerarin from healthy volunteers following oral administration of different doses of pueraria capsules [65]. In another investigation, the established ELISA method for geniposide detection was applied for assessment of biological samples from mice and pharmacokinetics of geniposide in mice after oral administration of Huanglian-Jiedu-Tang (a traditional Chinese medicine prescription) of three dosages [15]. 

Utilizing an established ELISA method, G-Re concentrations in the saliva of six healthy adults was also determined after the oral administration of a ginseng capsule to study the pharmacokinetics of G-Re in human saliva [8].

Zhao et al. generated a new anti-paeoniflorin monoclonal antibody and developed a sensitive and specific ELISA to efficiently measure the concentration of paeoniflorin, and then applied it to explore the pharmacokinetics of paeoniflorin in the presence of GA at different doses [13]. 

### 5.2. Fluorescence-Linked Immunosorbent Assay (FLISA)

FLISA has multiple benefits, including minimal pretreatment of samples prior to the assay, as well as being inexpensive, highly specific, and sensitive [66]. Compared with conventional ELISA, FLISA is a time-saving method because the time-consuming enzyme-substrate reaction necessary for ELISA can be avoided. Moreover, samples are exposed to environmental temperatures for shorter periods of time in FLISA, which minimizes the error compared with ELISA [67]. 

#### 5.2.1. Labelled with Fluorescein Isothiocyanate (FITC)

The MAbs against paeoniflorin was labelled with FITC and then used to develop an indirect competitive FLISA (icFLISA), which can be successfully applied for the detection and quantification of paeoniflorin in medicines and biological samples [68].

In another study, FITC-labelled baicalin-MAbs were used to develop an icFLISA to detect the baicalin (BAL) content in traditional Chinese medicine. This icFLISA for BAL is simple, rapid, and sensitive, with a 390-fold larger linear range and a two-fold lower limit of detection (LOD) compared with the previously developed ELISA [69]. 

#### 5.2.2. Labelled with Green Fluorescent Protein

Fluorescent single-domain antibodies (fluobodies), fusion proteins of a green fluorescent protein, and a single-chain variable fragment antibody (scFv) against plumbagin (PL) were produced by Sakamoto et al. and applied to the development of a rapid, sensitive, and simple icFLISA for detecting/determining PL in plant samples. The limit of detection for PL measurement in icFLISA (24 ng·mL^−1^) was improved to eight-fold higher than that in conventional ELISA (0.2 μg·mL^−1^) [67]. The similar recombinant fluobody against G-Re was also successfully constructed, expressed, and applied to icFLISA [70].

### 5.3. Eastern Blotting (Chromatographic Immunostaining)

Based on the application of MAbs, Eastern blotting (EB, developed and named by Shoyama et al.) [71,72] is an improved Western blotting technique, which makes use of a novel immunostaining methodology that permits detection and visualization of naturally-occurring bioactive compounds with low molecular weights, such as natural glycosides. MPNP with small molecules are easily washed out by buffer solutions during the immunostaining process without fixation. To overcome this shortcoming and facilitate fixation to the membrane, EB techniques employ a modified carboxyl activation method. The advantages of this new approach over the HPLC method are, mainly, its better cost-performance ratio (e.g., organic solvents and analytical equipment), speed, ease of use, and environmental friendliness, which are useful if large numbers of small samples are to be analyzed [18]. 

Depending on the categories of membranes, the EB technique can be classified into two major types.

#### 5.3.1. On Polyvinylidene Fluoride (PVDF) Membrane

PVDF membrane has been commonly used in Western blotting as a means of detecting protein targets [73]. With some modifications on the methodology, it was utilized in EB for analyzing MPNP.

##### Single Staining

Taking advantage of EB, a method for detecting glucuronides of GA was investigated and was applied to the immunocytolocalization of GA in *Glycyrrhiza* organs was investigated [74].

TLC-PVDF immunostaining was established for solasodine, which showed to be more sensitive compared to usual TLC staining with sulfuric acid or dragendorff reagents [71]. 

Fukuda et al. also succeeded in performing the EB of G-Rb1 using anti-G-Rb1 MAbs, which resulted in the staining of G-Rc, -Rd, -Re, and -Rg1 [75].

##### Double Staining

Different from single staining, the double staining method makes use of two distinct MAbs in one experiment, through which the accuracy of the immunostaining assay is being enhanced.

Fukuda et al. established a new double staining method for ginsenosides using anti-G-Rb1 and -G-Rg1 MAbs. This system enhanced the separate staining of ginsenosides having protopanaxatriol or protopanaxadiol in a molecule as an aglycon, making it possible to suggest which aglycon is attached and how many sugars are combined, leading to the structure of ginsenosides [76]. Such a double staining method was also established by Tanaka et al., but was combined with random amplified polymorphic DNA (RAPD) in order to standardize the quality of ginsengs [77].

In another case, leaf samples from *Cassia* species were analyzed using the double staining system employed MAbs against sennoside A (SA) and sennoside B (SB). SA and SB were unmistakably detected, whilst the detection of others sennosides was weaker [78]. This EB method is a useful approach for the identification of SA and SB in a background containing a large amount of impurities.

#### 5.3.2. On Polyether Sulfone (PES) Membrane

PES membrane is widely used for ultrafiltration systems [79,80] and enzyme immobilization units [81]. Recently, positively-charged PES membranes were found to be suitable for immunoblotting [82], and has been widely used in immunostaining techniques since then. 

##### Single Staining

The first EB on a positively-charged PES membrane was reported in 2005, which was applied to the immunoassay of glycyrrhizin [83]. Fujii et al. also developed a simple and specific initial screening assay system for GA using EB, and monitored GA concentrations in the plants over two years to confirm the homogeneity of GA concentrations in the cultivated plants. In all, 1025 plants were analyzed, and the highest concentration of GA was 5.36 percent dry weight (dw %) [64].

AA-I and AA-II in various samples derived from different parts of *Aristolochia* species and three related herbs free of aristolochic acid, but easily mistaken in the herb market, were visually detected using an EB method on PES membranes. The result showed that such an immunohistolocalization assay using anti-AA-Iand AA-II MAbs can distinguish *Aristolochia* species easily from other herbs containing no AAs [28].

The production of MAbs against G-Re [52] and G-Rb1 [84] and their application to the chromatographic immunostaining method for G-Re utilizing positively-charged PES membranes was also investigated. Due to the avoidance of low transfer efficiency from TLC to the PVDF membrane, these newly established methods enable the quantitative analysis of G-Re and G-Rb1 with the aid of NIH image analysis software. The same EB method for paeoniflorin [85] and saikosaponin [86] was established as well.

##### Double Staining

The establishment of a double EB by immunostaining using anti-GA and anti-liquiritin (Liq) MAbs was successfully applied in determining the immunohistochemical distribution and localization of GA and Liq in fresh licorice root. Moreover, its application to identify GA and Liq in licorice and Kampo medicines was also reported [87]. 

### 5.4. Sandwich ELISA

Among the immunoassay techniques, sandwich ELISA exhibits higher specificity, lower cross-reactivity, and a wider working range compared to the corresponding competitive assays [88]. However, for the reason that sandwich ELISA needs two distinct antibodies that can simultaneously bind to two antigen-binding sites on the desired analyte, such an immunoassay is difficult to be implemented on molecules with a molecular weight of less than 1000 Da. 

**Figure 2:**
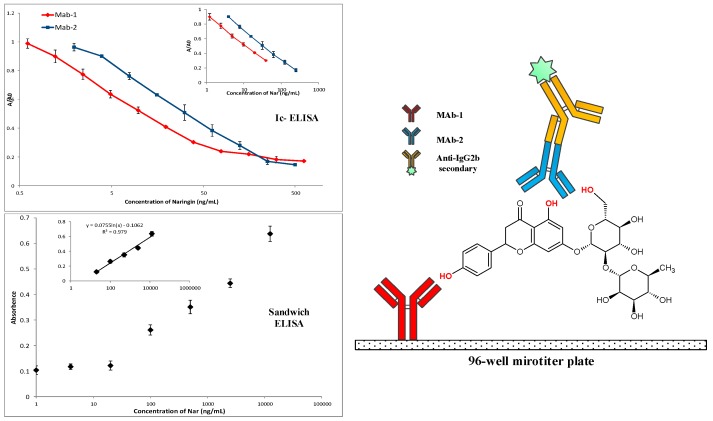
A true sandwich Enzyme-Linked Immunosorbent Assay (ELISA) for naringin and the comparison with ELISA [89]. Reprinted with permission from Huihua Qu et al. (2015), Copyright 2015 Analytica Chimica Acta.

Our team successfully developed, for the first time, a sandwich ELISA for Nar (Figure 2) providing an improved analytical approach with a broader detection range and higher precision compared to the icELISA utilizing each anti-Nar MAbs separately [89]. It is considered to be the only sandwich ELISA established for MPNP.

### 5.5. Immunochromatographic Assay

Immunochromatographic assay (ICA) using MAbs are highly specific and, therefore, useful for both semi-quantitative and sample screening [90]. Featuring the non-requirement of handling reagents and based on competitive immunoassays that utilize the antigen-antibody binding properties, such immunochromatographic assays provide sensitive detection of analytes and accelerate the analytical procedure. 

The quality of herbs and herbal products are of crucial importance for clinical efficacy. Therefore, rapid, sensitive, and low-cost detection of active or index components of herbal medicines are sorely needed for quality control and supervision of herbal products, ranging from the process of production, to circulation fields, to use. 

Although official drug-regulatory agencies and their affiliated analytical laboratories contraposing plant-derived medicinal products have been established, the capability to conduct surveillance still shows an insufficiency in confronting the tremendous pharmaceuticals market, which makes rapid testing technology and systems a pressing demand.

**Figure 3:**
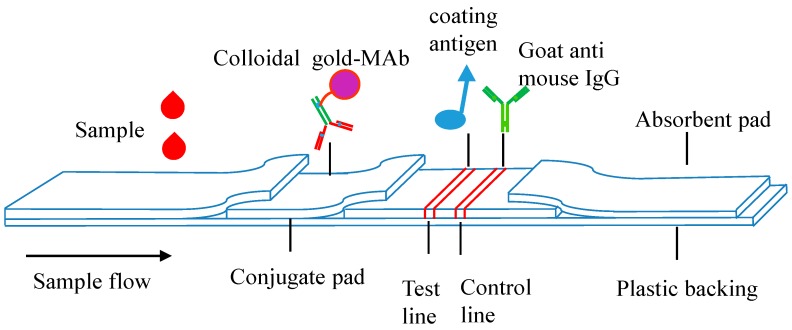
Schematic view of the lateral flow strip [91] Reprinted with permission from Huihua Qu et al. (2015), Copyright 2015 Biosensors and Bioelectronics.

Based on the specific MAbs and lateral flow technology (Figure 3), MPNP immunochromatographic test strips (ITS) using various materials as labels, such as colloidal gold and fluorescent quantum dots, can have greater sensitivity (Table 4), and achieve continuous, in situ, visualized, rapid measurement and quality control of MPNP, thus providing a feasible pathway to the establishment of rapid testing systems for herbal medicine and products, and break away from the restrictions of large-scale equipment and professional staff requirements.

Additionally, the combination of the ICA and other immunoassay methods, such as ELISA, will also provide a robust means of MPNP analyses in a timely and efficient manner. 

#### 5.5.1. Labeled with Colloidal Gold

Paudel et al. developed a one-step immunochromatographic strip test which is useful as a simple and rapid screening procedure for the semi-quantitative detection of BAL in various materials. In addition, with the combination of this ICA and ELISA, they strengthened the accuracy of BAL analyses [92].

Sakamoto et al. established a one-step indirect competitive ICA for rapid and sensitive detection of total isoflavone glycosides (DA and genistin) using gold nanoparticles conjugated with a monoclonal antibody against DA [44].

Wan et al. described an immunochromatographic strip test based on an immunoassay system with MAbs against G-Rb1 and G-Rg1 together. It can be used to detect G-Rb1 and G-Rg1 (two major components in ginseng) on a single strip simultaneously. Such an assay can be conducted on-site where ginseng samples are collected [82].

Putalun et al. also developed a one-step immunochromatographic strip test for the detection of SA and SB, which have the characteristic of high sensitivity (detection limits: 125 ng/mL) [93]. 

#### 5.5.2. Labeled with Quantum Dots

Recently, an exciting new nanomaterial, quantum dots (QDs), emerged on the scene of biosensors. The features of size-tunable emission, broad absorption, intense brightness, narrow emission spectra, and exceptional resistance to photobleaching have made QDs more attractive than traditional colloidal gold or fluorescent probes for developing analytical applications [94]. ITS integrated with QDs as fluorescence labels could provide more rapid, accurate, sensitive qualitative or quantitative detection of MPNPs.

**Figure 4:**
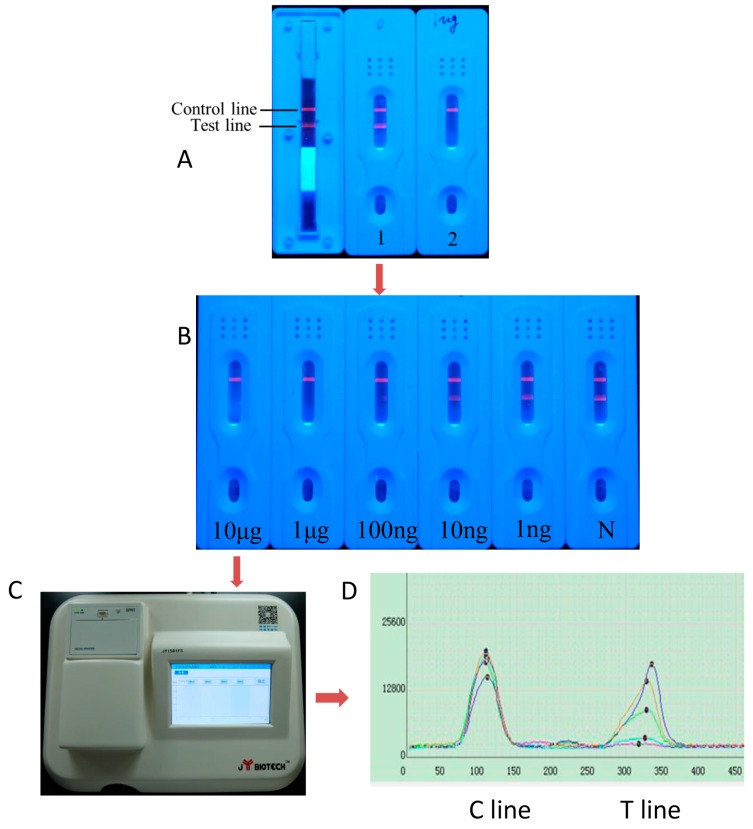
Characterization of the lateral flow immunoassay for PUE. (**A**) Results obtained using the ITS test for puerarin showing negative (**A1**) and positive (**A2**) samples in ultraviolet light. Fluorescence appeared at both the test and control lines if a sample was free of puerarin. Fluorescence appeared only at the control line if a sample was positive for puerarin; (**B**) Photographs of results for standard solutions containing different concentrations of puerarin assayed using the ICS; (**C**) Photograph of the matched scanning luminoscope; (**D**) The fluorescent intensity pattern scanned by the luminoscope [91].

QDs-based lateral flow biosensors have been widely used in clinical diagnosis and daily life for analytes, such as proteins [95], pathogens [96], and antibiotics [97], but are still in their infancy in terms of MPNP detection. In a previous study [91], a rapid (within 10 min) quantitative lateral-flow immunoassay using a QD-antibody probe was developed for the analysis of puerarin in water and biological samples (Figure 4). This represents a low-cost, on-site, and user-friendly method for detecting puerarin in plant materials and biological samples. To the best of our knowledge, this is, to date, the first and the only report of the quantitative detection of MPNPs by QD-based immunochromatography. 

As shown in Table 4, QD-based immunochromatography possesses lower detection limits compared to other labels and represents higher sensitivity, which is an obvious advantage for the detection of MPNPs, especially for trace components, in herbal products. Therefore, such technology contributes to the trends and future of MPNP detection and study. 

### 5.6. Immunoaffinity Chromatography Column

**Figure 5:**
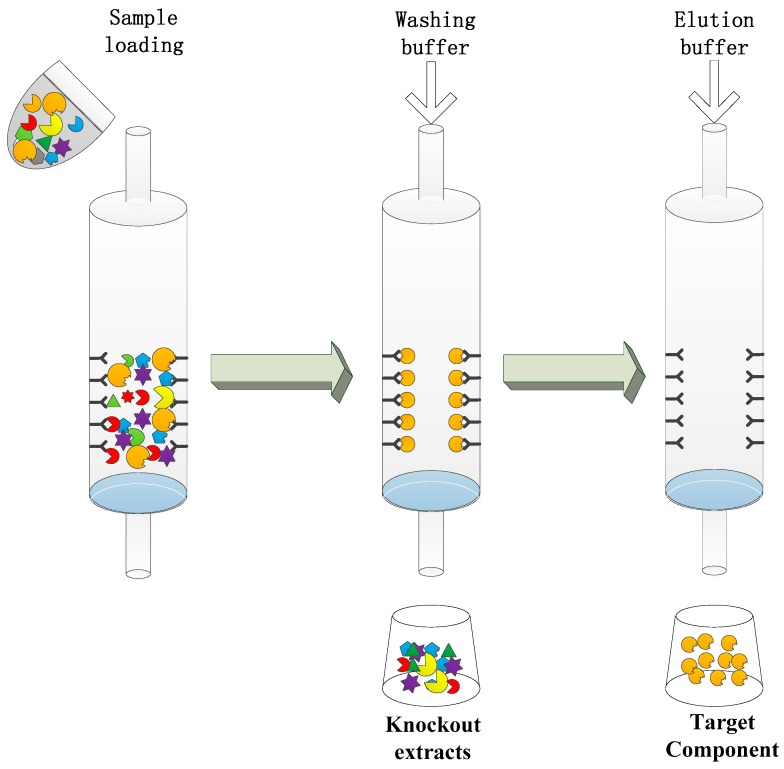
Schematic of the immunoaffinity column.

Generally, strategies for detecting and separating MPNPs have been based on HPLC and chromatographic techniques, which are tedious and time-consuming procedures. Taking advantage of the specific and reversible interactions between antibodies and their cognate antigens, an immunoaffinity (IA) column (Figure 5) enables the isolation of a target compound with high efficiency and purity, which has been proved by high-performance liquid chromatography fingerprints and high-performance liquid chromatography with mass spectrometry [98]. Definitely, IA chromatography consumes a larger amount of MAbs than do immunochemical detection techniques. However, finishing with a proper wash, and equilibrated after each partial use, the IA column can be re-used. Therefore, once an IA column is prepared, it may be used several times without a loss in activity.

The application of an IA chromatography column can simplify the pretreatment and the isolation process greatly compared to conventional methods, providing a potential method for extracting the target MPNP from structurally-similar compounds, even chiral compounds, in natural products. (Table 5). It could also be used as a separation technique for analyte enrichment and/or cleanup for subsequent pharmacological analysis and drug discovery. 

Furthermore, fingerprint techniques have now been widely used in the quality control and supervision of plant-derived medicinal products, including medicinal materials and medicine prescriptions, through the qualitative and quantitative detection of one or more specific index component [99]. Through the high specificity of MAbs-MPNP interactions, an IA column is particularly suited to selective separation of the target component from other contaminants in the drug sample, especially with respect to the separation challenge in complex sample media. Being employed as a rapid and simple cleanup method previously, an IA column can be used in conjunction with conventional instrumental (i.e., GC, GC/MS, HPLC, LC/MS) detection, which will avoid tedious sample preparation procedures to some degree, and thereby significantly facilitate the plant-derived medicinal product’s supervision system.

#### 5.6.1. Rapid Separation and Purification of Target Compounds

Qu et al. prepared an IA column using the generated MAbs against DA, which can efficiently and specifically extract DA, glycitein, and genistin from numerous structurally-similar soy isoflavones in leguminous plants, thereby providing a new method for the extraction of target components from similar compounds in natural products [45]. 

Another IA column was obtained by coupling anti-Nar MAbs to CNBr-activated Sepharose 4B and a rapid IA chromatography assay for Nar was developed. It was used to separate Nar *from Citrus aurantium*, and the IA column can efficiently and specifically capture approximately 250 μg of Nar without cross-reacting with its structurally-similar compounds [14]. Additionally, rapid separation of forskolin [100], solasodine glycosides [101], puerarin [12], G-Rb1 [75], and GA [11] was successively established by the IA column as well.

Interestingly, taking advantage of a generated anti-G-Rh1 antibody, a new IA column was provided which could be employed as a promising approach and method for the resolution of 20(*S*)-Rg2 and 20(*R*)-Rg2 (a pair of epimers of G-Rg2), due to its high cross-reactivity with the former but no cross-reactivity with the the latter. However, although this study demonstrates the potential of the IA column for separation of 20(*S*)- and(*R*)-type-ginsenosides, the columns used are only capable of separating a limited amount of the epimers [9]. Such findings provides a totally new thought and potential method of using the IA column for the identification and separation of epimers, which is a challenge for modern analysis techniques. 

#### 5.6.2. Bioactivity and Pharmacological Analysis

Herbal medicine (HM), especially its prescription, is a complex system. In the modern research of HM, simply focusing on one specific chemical compound is insufficient. In order to understand the pharmacological mechanisms and synergistic effects of HM, it is important to investigate the degree of the contribution of a particular component to the overall activity of a complex herbal mixture, and the connections between a principal component and a primary medicinal property. 

Through the IA chromatography column made by MAbs, knock out (KO) extract and the target compound can be obtained, respectively, consequently providing a useful approach for determination of not only the real pharmacological functions of natural compounds and phytochemical mixtures, but also the synergistic function. This is an ideal way to identify the biological role of major components in plant or their derived prescriptions. The new method can reflect not only the overall combined pharmacological effects of HM but also the effect of individual components. It is an effective way to explain the degree of contribution of one specific component to the overall activity of an HM prescription.

Yuan et al. applied the IA chromatography column for preparation of American ginseng berry extract (AGBE) without G-Re, and then validated the anti-diabetic and anti-obesity effects of the AGBE and GRe-KO-AGBE extracts [102]. 

Uto et al. prepared GA-removed extract (GA-KO extract) from licorice extract (LE) using the IA column conjugated with anti-GA MAbs, and analyzed the effect on lipopolysaccharide-induced nitric oxide production. It was found that the treatment of GA alone could not show the suppression of NO production and inducible NO synthase (iNOS) expression, and the inhibitory effect of GA–KO extract was significantly attenuated compared with LE. However, the attenuated inhibition reappeared after the combined treatment with GA-KO extract and GA. These results indicate that GA may exert synergistic suppression of iNOS expression when coexisting with the other constituents contained in licorice extract [103].

An IA column made with an anti-BAL monoclonal antibody was able to specifically knock out BAL, oroxylin A-7-*O*-glucuronide, wogonoside, wogonin, and baicalein from a gegenqinlian decoction. The pharmacological analysis demonstrated that the gegenqinlian decoction and its knocked-out fraction showed no significant difference, which indicated that the BAL, and all the other components that were knocked out by the IA column, might not be key compounds for the induction of gegenqinlian decoction superoxide dismutase secretion [98].

## 6. Conclusions and Future Prospects

In summary, monoclonal antibodies are tools of vital importance in modern bioscience research, and have been greatly and rapidly developed in recent years in MPNP research. Various immunoassay methods established on the basis of these MPNP MAbs has covered the wide phytochemical fields from quality control of HM, to medicinal plant breeding regarding bioactive components, to the pharmacological analysis. 

Certainly, if the field of MPNP immunoassay is to remain a major analytical tool, the technologies and materials upon which it is based must continually improve. At the same, the numbers of MPNP MAbs need to continue to grow for the extension of the library. It is also worth thinking about how to integrate immunoassay methods into existing MPNP analytical tools and facilities and leveraging the benefits of them to provide a synergistic effect, consequently to better serve the area of MPNP research and detection. These all have great meaning for the development of phytopharmacy. Moreover, some newly emerging antibody types, such as single-domain antibody (nanobody), and technologies have been applied in immunoassay research recently [104,105]. In the future, these new antibody technologies could also be used or MAbs production and research of MPNP. 

In the future, MAbs against MPNP and immunoassays depend on a broad application prospect in the field of receptor binding analysis, drug discovery, rapid detection of target compounds, and quantitative and/or qualitative analytical techniques of MPNP research. Additionally, it is interesting to note that materials science is reaching into the nano realm. Innovative interfaces between new materials represented by QDs and MPNP will be a driver in rapid detection technologies development for years to come, and they are also expected to bring advances to the area of not only novel rapid detection opportunities, but also in vitro and in vivo imaging and tracking for MPNP.

## Figures and Tables

**Table 1:** Coupling methods between carrier protein and medical plant-derived natural products (MPNP) that were applied in the synthesis of MPNP artificial antigens.

Coupling Method	MPNP	Reference
Sodium periodate oxidation method	Ginsenoside Re	[8]
	Ginsenoside Rh1	[9]
	Ginkgolic Acids	[10]
	Glycyrrhizic acid	[11]
	Puerarin	[12]
	Paclitaxel	[7]
	Paeoniflorin	[13]
	Naringin	[14]
	Geniposide	[15]
	Solamargine	[16]
	Crocin	[17]
	Saikosaponins a	[18]
	Liquiritin	[19]
	Bacopaside I	[20]
	Notoginsenoside R1	[21]
	Ginsenoside Rg1	[22]
	Ginsenoside Rb1	[23]
	Daidzin	[24]
Carbodiimide method	Sennoside B	[25]
	Chenodeoxycholic acid	[26]
	Baicalin	[27]
	Aristolochic Acid-I	[28]
	Aristolochic Acid-II	[29]
	Artemisinin	[30]
	Coptisine	[31]
	Berberine	[32]
	Glycyrrhetic acid	[33]
	Forskolin	[34]
	Mitragynine	[35]
Mixed anhydrides reaction	Hyodeoxycholic Acid	[36]
Mannich condensation	Daidzin	[37]
Succinate method	Plumbagin	[38]
	Aconitine	[39]
Mctive ester method	Tetrahydrocannabinolic-Acid	[40]

**Table 2:** Existing anti-MPNP monoclonal antibodies (MAbs).

Classification	MAbs	Structure	Reference
Alkaloid	Tetrahydrocannabinolic acid	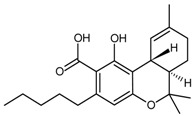	[40]
	Solasodine	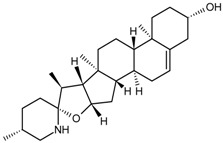	[48]
	Solamargine	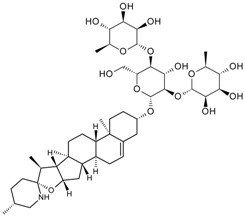	[16]
	Mitragynine	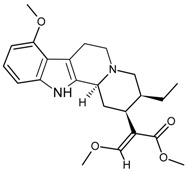	[35]
	Mconitine	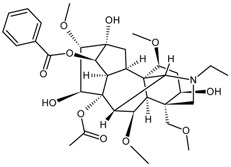	[39]
	Coptisine	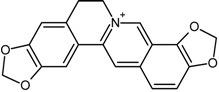	[31]
	Berberine	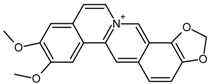	[32]
Monoterpene	Paeoniflorin	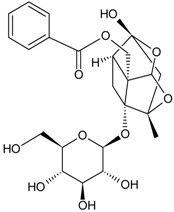	[13]
Sesquiterpenes	Artemisinin	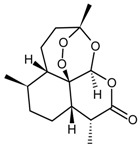	[30]
Diterpene	Forskolin	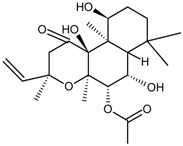	[34]
	Paclitaxel	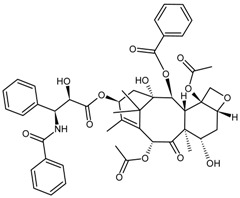	[7]
Triterpene	Ginsenoside Rb1	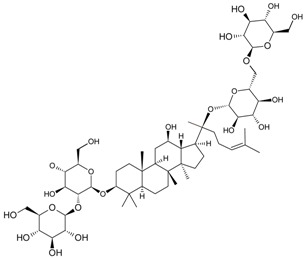	[23]
	Bacopaside I	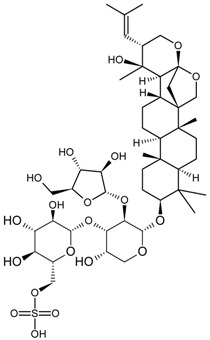	[20]
	Saikosaponin a	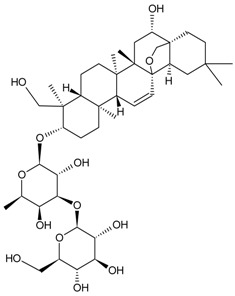	[18]
	Glycyrrhizic acid	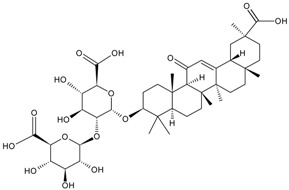	[11]
	Ginsenoside Rg1	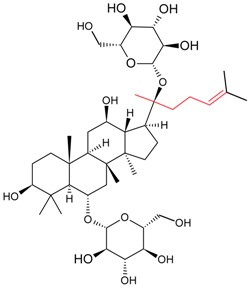	[22]
	Ginsenoside Re	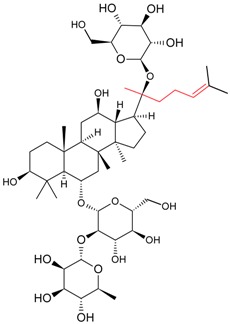	[8]
	Ginsenoside Rh1	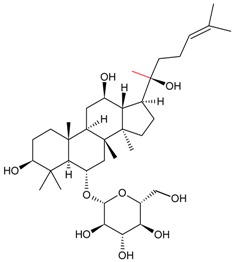	[9]
	Notoginsenoside R1	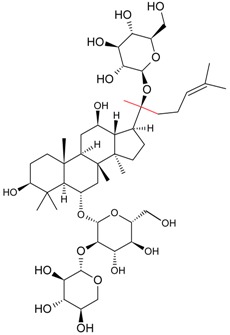	[21]
Tetraterpene	Crocin	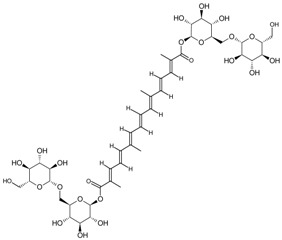	[17]
Iridoid	Geniposide	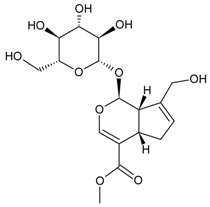	[15]
Quinones	Sennoside B	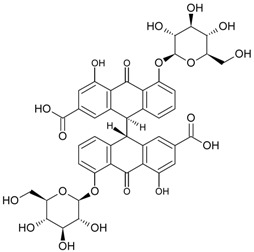	[25]
	Plumbagin	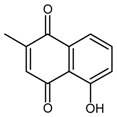	[38]
	Aristolochic Acid I	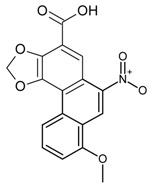	[28]
	Aristolochic Acid II	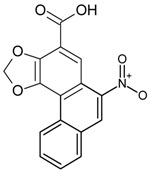	[29]
Flavonoid	Naringin	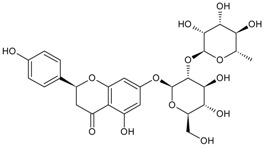	[14]
	Baicalin	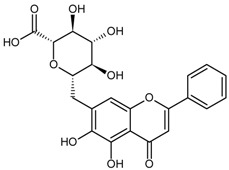	[27]
	Liquiritin	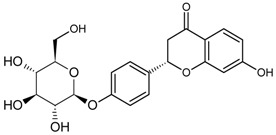	[19]
	Daidzin	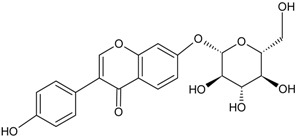	[45]
	Puerarin	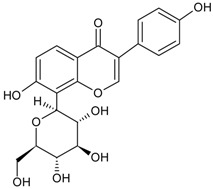	[12]
Bile acids	Chenodeoxycholic acid	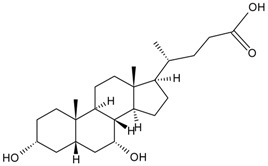	[26]
	Hyodeoxycholic acid	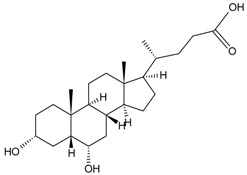	[36]
Phenolic acids	Ginkgolic Acid	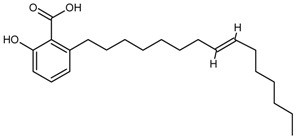	[10]

**Table 3:** Cross-reactivity (%) of anti-MPNP MAbs (related compounds with cross-reactivity greater than 2% were extracted from the original references and displayed in this table). The cross-reactivity of the MAbs against various compounds was evaluated using the Weiler and Zenk’s equation [50]: $\mathrm{Cross}-\mathrm{reactivity}\;\left(\%\right)=\frac{{\mathrm{IC}}_{50}\;\mathrm{for}\;\mathrm{the}\;\mathrm{target}\;\mathrm{compound}}{{\mathrm{IC}}_{50}\;\mathrm{for}\;\mathrm{related}\;\mathrm{compound}\;\mathrm{under}\;\mathrm{investigation}}\times 100$.

MAbs	Compound	Cross-Reactivity (%)	References
Forskolin	7-deacetyl-forskolin	5.60	[34]
Solamargine	Solasonine	92.1	[16]
	Solasodine	43.8	
Glycyrrhizic acid	Glycyrrhetinic acid	31.57	[11]
Glycyrrketic Acid	18αH-Olean-ll-oxo-12-ene-30-oic acid (18α-HGA)	18.9	[33]
	3α-hydroxy GA; Olean-12-ene-3α-hydroxy-ll-oxo-30-oic acid	2.3	
	30-ol GA; Olean-12-ene-11-ox03β	3.66	
Ginsenoside Rg1	Ginsenoside Re	3.3	[22]
Crocin	Crocetin triglucoside	39.6	[17]
	Crocetin diglucoside	26.8	
	Crocetin	2.6	
Sennoside B	Sennoside A	2.45	[25]
Paeoniflorin	Albiflorin	143.7	[51]
	Oxypaeoniflorin	5.2	
	Benzoylpaeoniflorin	29.4	
Saikosaponin a	Saikosaponin c	2.65	[18]
	Saikosaponin d	3.76	
Ginkgolic Acids	Olivetolic acid	54.2	[10]
	Divarinolic acid	9.98	
Coptisine	Berberine	20.6	[31]
	Palmatine	20.4	
	Berberrubine	29.8	
	Jateorrhizine	14	
Berberine	Coptisine	140.7	[32]
	Palmatine	50.7	
	Berberrubine	15.1	
	9-Acetylberberine	12.3	
Ginsenoside Re	Ginsenoside Rg1	70.94	[52]
	Ginsenoside Rd	76.23	
Bacopaside I	Bacopaside II	299.33	[20]
	Bacopasaponin C	64.05	
	Bacopaside V	94.4	
Artemisinin	Artesunate	630	[30]
	Dihydroartemisinin	29.9	
Plumbagin	Menadione	80.50	[38]
Baicalin	Baicalein	51.41	[53]
Aristolochic acid-II	Aristolochic acid I	3.45	[29]
	AA-IIIa	17	
Aristolochic acids-I	AA-II 69.3.02.	69.3	[28]
	Decarboxy-AA-II	8.9	
Aconitine	Mesaconitine	93.1	[39]
	Hypaconitine	104	
	Jesaconitine	65.6	
	Benzoylaconine	8.84	
	Benzoylmesaconine	4.96	
Mitragynine	Speciogynine	30.54	[35]
	Paynantheine	24.83	
	Mitraciliatine	8.63	
	Tryptamine	2.79	
Notoginsenoside R1	Ginsenoside Rb1	2.61	[21]
Paclitaxel	Docetaxel	70.7	[54]
	7-Xylosyltaxol	31.8	
liquiritin	Liquiritigenin	33.09	[19]
Daidzin (SPOM)	daidzein	16.16	[24]
	Genistin	82.35	
	Genistein	24.42	
	Glycitin	11.92	
Daidzin (Mannich condensation)	Daidzein	1.57	[37]
Naringin	Neohesperidin	18.80	[14]
Puerarin	Baicalein	58.1	[12]
Chenodeoxycholic acid	Cholic acid	2.1	[26]
	Deoxycholic acid	4.3	
Ginsenoside-Rh1	Ginsenoside Rg2(*S*)	470.65	[9]
	Ginsenoside Rg3(*S*)	13.88	
	Ginsenoside Rh2(*S*)	12.25	

**Table 4:** The detection limits of established immunochromatographic assay using anti-MPNP MAbs.

MAbs	Label	Detection Limits (μg/mL)	References
Sennoside B	Colloidal gold	0.1250	[93]
Rb1, Rg1	Colloidal gold	2.0000	[82]
Glycyrrhizic acid	Colloidal gold	0.2500	[90]
Baicalin	Colloidal gold	0.6000	[92]
Daidzin	Colloidal gold	0.1250	[44]
Puerarin	CdSe/ZnS QDs	0.0058	[91]

**Table 5:** The conjugate capacity between anti-MPNP MAbs and the carriers of the immunoaffinity (IA) column.

Analyte	Capacity of Immunoaffinity Chromatography Column	References
Forskolin	9.41 ug/mL gel	[100]
Solamargine	6.19μg/mL gel	[101]
Ginsenoside Rb1	20 mg/mL gel	[75]
Glycyrrhizin	33.5 μg/mL gel	[49]
Puerarin	22 μg/mL gel	[12]
Daidzin	12.26 mg/g Sepharose 4B	[45]
Naringin	10 mg/g Sepharose 4B	[14]

Analyte

Capacity of Immunoaffinity Chromatography Column

References

## References

[1] De Smet P.A. (2002). Herbal Remedies. N. Engl. J. Med..

[2] Osbourn A.E., Lanzotti V. (2009). Plant-Derived Natural Products: Synthesis, Function, and Application.

[3] Rowinsky E.K., Donehower R.C. (1993). The Clinical Pharmacology Of Paclitaxel (Taxol). Semin. Oncol..

[4] Klayman D.L., Lin A.J., Acton N., Scovill J.P., Hoch J.M., Milhous W.K., Theoharides A.D., Dobek A.S. (1984). Isolation of Artemisinin (Qinghaosu) from Artemisia Annua Growing in the United States. J. Nat. Prod..

[5] Kohler G., Milstein C. (1975). Continuous Cultures of Fused Cells Secreting Antibody of Predefined Specificity. Nature.

[6] Van Emon J.M. (2007). Immunoassay and Other Bioanalytical Techniques.

[7] Leu J.G., Chen B.X., Schiff P.B., Erlanger B.F. (1993). Characterization of Polyclonal and Monoclonal Anti-Taxol Antibodies and Measurement of Taxol in Serum. Cancer Res..

[8] Qu H., Sai J., Wang Y., Sun Y., Zhang Y., Li Y., Zhao Y., Wang Q. (2014). Establishment of an Enzyme-Linked Immunosorbent Assay and Application on Determination of Ginsenoside Re in Human Saliva. Planta Med..

[9] Qu H., Wang Y., Shan W., Zhang Y., Feng H., Sai J., Wang Q., Zhao Y. (2015). Development of Elisa for Detection of Rh1 and Rg2 and Potential Method of Immunoaffinity Chromatography for Separation of Epimers. J. Chromatogr. B Anal. Technol. Biomed. Life Sci..

[10] Loungratana P., Tanaka H., Shoyama Y. (2004). Production of Monoclonal Antibody against Ginkgolic Acids in Ginkgo Biloba Linn. Am. J. Chin. Med..

[11] Zhang Y., Qu H., Zeng W., Zhao Y., Shan W., Wang X., Wang Q., Zhao Y. (2015). Development of an Enzyme-Linked Immunosorbent Assay and Immunoaffinity Chromatography for Glycyrrhizic Acid Using an Anti-Glycyrrhizic Acid Monoclonal Antibody. J. Sep. Sci..

[12] Qu H., Zhang G., Li Y., Sun H., Sun Y., Zhao Y., Wang Q. (2014). Development of an Enzyme-Linked Immunosorbent Assay Based on Anti-Puerarin Monoclonal Antibody and Its Applications. J. Chromatogr. B Anal. Technol. Biomed. Life Sci..

[13] Zhao Y., Qu H., Wang X., Zhang Y., Shan W., Zhao Y., Wang Q. (2015). A Sensitive and Specific Indirect Competitive Enzyme-Linked Immunosorbent Assay for Detection of Paeoniflorin and Its Application in Pharmacokinetic Interactions between Paeoniflorin and Glycyrrhizinic Acid. Planta Med..

[14] Qu H., Zhang Y., Qu B., Cheng J., Liu S., Feng S., Wang Q., Zhao Y. (2016). Novel Immunoassay and Rapid Immunoaffinity Chromatography Method for the Detection and Selective Extraction of Naringin in Citrus Aurantium. J. Sep. Sci..

[15] Qu H.H., Sun Y., Wu T.T., Zhang G.L., Cheng J.J., Wang X.Q., Feng H.B., Zhao Y., Wang Q.G. (2014). Pharmacokinetics of Geniposide by Monoclonal Antibody-Based Icelisa in Mice after Oral Administration of Huanglian-Jiedu-Tang. Biol. Pharm. Bull..

[16] Ishiyama M., Shoyama Y., Murakami H., Shinohara H. (1995). Production of Monoclonal Antibodies and Development of an Elisa for Solamargine. Cytotechnology.

[17] Xuan L., Tanaka H., Xu Y., Shoyama Y. (1999). Preparation of Monoclonal Antibody against Crocin and Its Characterization. Cytotechnology.

[18] Zhu S., Shimokawa S., Shoyama Y., Tanaka H. (2006). A Novel Analytical Elisa-Based Methodology for Pharmacologically Active Saikosaponins. Fitoterapia.

[19] Fujii S., Morinaga O., Uto T., Nomura S., Shoyama Y. (2014). Development of a Monoclonal Antibody-Based Immunochemical Assay for Liquiritin and Its Application to the Quality Control of Licorice Products. J. Agric. Food Chem..

[20] Phrompittayarat W., Putalun W., Tanaka H., Jetiyanon K., Wittaya-Areekul S., Ingkaninan K. (2007). Determination of Pseudojujubogenin Glycosides from Brahmi Based on Immunoassay Using a Monoclonal Antibody against Bacopaside I. Phytochem. Anal. PCA.

[21] Limsuwanchote S., Wungsintaweekul J., Yusakul G., Han J.Y., Sasaki-Tabata K., Tanaka H., Shoyama Y., Morimoto S. (2014). Preparation of a Monoclonal Antibody against Notoginsenoside R1, a Distinctive Saponin from Panax Notoginseng, and Its Application to Indirect Competitive Elisa. Planta Med..

[22] Fukuda N., Tanaka H., Shoyama Y. (2000). Formation of Monoclonal Antibody against a Major Ginseng Component, Ginsenoside Rg1 and Its Characterization. Monoclonal Antibody for a Ginseng Saponin. Cytotechnology.

[23] Tanaka H., Fukuda N., Yahara S., Isoda S., Yuan C.S., Shoyama Y. (2005). Isolation of Ginsenoside Rb1 from Kalopanax Pictus by Eastern Blotting Using Anti-Ginsenoside Rb1 Monoclonal Antibody. Phytother. Res. PTR.

[24] Sakamoto S., Yusakul G., Pongkitwitoon B., Paudel M.K., Tanaka H., Morimoto S. (2015). Simultaneous Determination of Soy Isoflavone Glycosides, Daidzin and Genistin by Monoclonal Antibody-Based Highly Sensitive Indirect Competitive Enzyme-Linked Immunosorbent Assay. Food Chem..

[25] Morinaga O., Nakajima S., Tanaka H., Shoyama Y. (2001). Production of Monoclonal Antibodies against a Major Purgative Component, Sennoside B, Their Characterization and Use in Elisa. Analyst.

[26] Zhang Y., Qu H., Feng H., Wang X., Shan W., Zeng W., Wang Q., Zhao Y. (2015). Development of an Enzyme-Linked Immunosorbent Assay for Chenodeoxycholic Acid Using an Anti-Chenodeoxycholic Acid Monoclonal Antibody. Anal. Methods.

[27] Zhao Y., Kong H., Sun Y., Feng H., Zhang Y., Su X., Qu H., Wang Q. (2014). Assessment of Baicalin in Mouse Blood by Monoclonal Antibody-Based Icelisa. Biomed. Chromatogr. BMC.

[28] Williams M., Malick J.B. (2013). Drug Discovery and Development.

[29] Tian M., Tanaka H., Shang M.Y., Karashima S., Chao Z., Wang X., Cai S.Q., Shoyama Y. (2008). Production, Characterization of a Monoclonal Antibody against Aristolochic Acid-Ii and Development of Its Assay System. Am. J. Chin. Med..

[30] Tanaka H., Putalun W., De-Eknamkul W., Matangkasombut O., Shoyama Y. (2007). Preparation of a Novel Monoclonal Antibody against the Antimalarial Drugs, Artemisinin and Artesunate. Planta Med..

[31] Kim J.S., Tanaka H., Yuan C.S., Shoyama Y. (2004). Development Of Monoclonal Antibody against Isoquinoline Alkaloid Coptisine and Its Application for the Screening of Medicinal Plants. Cytotechnology.

[32] Kim J.S., Tanaka H., Shoyama Y. (2004). Immunoquantitative Analysis for Berberine and Its Related Compounds Using Monoclonal Antibodies in Herbal Medicines. Analyst.

[33] Mizugaki M., Itoh K., Hayasaka M., Ishiwata S., Nozaki S., Nagata N., Hanadate K., Ishida N. (1994). Monoclonal Antibody-Based Enzyme-Linked Immunosorbent Assay for Glycyrrhizin and Its Aglycon, Glycyrrhetic Acid. J. Immunoass..

[34] Sakata R., Shoyama Y., Murakami H. (1994). Production of Monoclonal Antibodies and Enzyme Immunoassay for Typical Adenylate Cyclase Activator, Forskolin. Cytotechnology.

[35] Limsuwanchote S., Wungsintaweekul J., Keawpradub N., Putalun W., Morimoto S., Tanaka H. (2014). Development of Indirect Competitive Elisa for Quantification of Mitragynine in Kratom (*Mitragyna Speciosa* (Roxb.) Korth.). Forensic Sci. Int..

[36] Zhao Y., Qu H., Zhang Y., Sun Y., Feng H., Shan W., Zhao Y., Wang Q. (2015). Enzyme-Linked Immunosorbent Assay for Hyodeoxycholic Acid in Pharmaceutical Products Using a Monoclonal Antibody. Anal. Lett..

[37] Yusakul G., Sakamoto S., Juengwatanatrakul T., Putalun W., Tanaka H., Morimoto S. (2016). Preparation and Application of a Monoclonal Antibody against the Isoflavone Glycoside Daidzin Using a Mannich Reaction-Derived Hapten Conjugate. Phytochem. Anal. PCA.

[38] Sakamoto S., Putalun W., Tsuchihashi R., Morimoto S., Kinjo J., Tanaka H. (2008). Development of an Enzyme-Linked Immunosorbent Assay (ELISA) Using Highly-Specific Monoclonal Antibodies against Plumbagin. Anal. Chim. Acta.

[39] Kido K., Edakuni K., Morinaga O., Tanaka H., Shoyama Y. (2008). An Enzyme-Linked Immunosorbent Assay for Aconitine-Type Alkaloids Using an Anti-Aconitine Monoclonal Antibody. Anal. Chim. Acta.

[40] Tanaka H., Goto Y., Shoyama Y. (1996). Monoclonal Antibody Based Enzyme Immunoassay for Marihuana (Cannabinoid) Compounds. J. Immunoass..

[41] Erlanger B.F., Beiser S.M. (1964). Antibodies Specific for Ribonucleosides and Ribonucleotides and Their Reaction With DNA. Proc. Natl. Acad. Sci. USA.

[42] Hermanson G.T. (2008). Bioconjugate Techniques.

[43] Goto Y.S.Y., Morimoto S., Shoyama Y. (1994). Determination of Tetrahydrocannabinolic Acid-Carrier Protein Conjugate by Matrix-Assisted Laser Desorption/Ionization Mass Spectrometry and Antibody Formation. Org. Mass Spectrom..

[44] Sakamoto S., Yusakul G., Pongkitwitoon B., Tanaka H., Morimoto S. (2016). Colloidal Gold-Based Indirect Competitive Immunochromatographic Assay for Rapid Detection of Bioactive Isoflavone Glycosides Daidzin and Genistin in Soy Products. Food Chem..

[45] Qu H., Qu B., Wang X., Zhang Y., Cheng J., Zeng W., Liu S., Wang Q., Zhao Y. (2016). Rapid, Sensitive Separation of the Three Main Isoflavones in Soybean Using Immunoaffinity Chromatography. J. Sep. Sci..

[46] Zhang C. (2012). Hybridoma Technology for the Generation of Monoclonal Antibodies.

[47] Al-Rubeai M. (2011). Antibody Expression and Production.

[48] Putalun W., Taura F., Qing W., Matsushita H., Tanaka H., Shoyama Y. (2003). Anti-Solasodine Glycoside Single-Chain Fv Antibody Stimulates Biosynthesis of Solasodine Glycoside in Plants. Plant Cell Rep..

[49] Xu J., Tanaka H., Shoyama Y. (2007). One-Step Immunochromatographic Separation and Elisa Quantification of Glycyrrhizin from Traditional Chinese Medicines. J. Chromatogr. B Anal. Technol. Biomed. Life Sci..

[50] Weiler E.W., Kruger H., Zenk M.H. (1980). Radioimmunoassay for the Determination of the Steroidal Alkaloid Solasodine and Related Compounds in Living Plants and Herbarium Specimens. Planta Med..

[51] Lu Z., Morinaga O., Tanaka H., Shoyama Y. (2003). A Quantitative Elisa Using Monoclonal Antibody to Survey Paeoniflorin and Albiflorin in Crude Drugs and Traditional Chinese Herbal Medicines. Biol. Pharm. Bull..

[52] Morinaga O., Tanaka H., Shoyama Y. (2006). Detection and quantification of ginsenoside Re in ginseng samples by a chromatographic immunostaining method using monoclonal antibody against ginsenoside Re. J. Chromatogr..

[53] Kido K., Morinaga O., Shoyama Y., Tanaka H. (2008). Quick Analysis of Baicalin in Scutellariae Radix by Enzyme-Linked Immunosorbent Assay Using a Monoclonal Antibody. Talanta.

[54] Chao Z., Tan M., Paudel M.K., Sakamoto S., Ma L., Sasaki-Tabata K., Tanaka H., Shoyama Y., Xuan L., Morimoto S. (2013). Development of an Indirect Competitive Enzyme-Linked Immunosorbent Assay (Icelisa) Using Highly Specific Monoclonal Antibody against Paclitaxel. J. Nat. Med..

[55] Bensoussan A. (2005). Herbal Medicine Safety.

[56] Bent S. (2008). Herbal Medicine in the United States: Review of Efficacy, Safety, and Regulation: Grand Rounds at University of California, San Francisco Medical Center. J. Gen. Intern. Med..

[57] Saad B., Azaizeh H., Abu-Hijleh G., Said O. (2006). Safety of Traditional Arab Herbal Medicine. Evid. Based Complement. Altern. Med..

[58] Balachandran P., Wei F., Lin R.C., Khan I.A., Pasco D.S. (2005). Structure Activity Relationships of Aristolochic Acid Analogues: Toxicity in Cultured Renal Epithelial Cells. Kidney Int..

[59] Cosyns J.P., Dehoux J.P., Guiot Y., Goebbels R.M., Robert A., Bernard A.M., van de Strihou C. (2001). Chronic Aristolochic Acid Toxicity in Rabbits: A Model of Chinese Herbs Nephropathy?. Kidney Int..

[60] Shang M.Y., Tian M., Tanaka H., Li X.W., Cai S.Q., Shoyama Y. (2011). Quality Control of Traditional Chinese Medicine by Monoclonal Antibody Method. Curr. Drug Discov. Technol..

[61] Morinaga O., Uto T., Sakamoto S., Tanaka H., Shoyama Y. (2009). Enzyme-Linked Immunosorbent Assay for Total Sennosides Using Anti-Sennside a and Anti-Sennoside B Monoclonal Antibodies. Fitoterapia.

[62] Tanaka H., Shoyama Y. (1999). Monoclonal Antibody Against Tetrahydrocannabinolic Acid Distinguishes Cannabis Sativa Samples from Different Plant Species. Forensic Sci. Int..

[63] Sritularak B., Morinaga O., Yuan C.S., Shoyama Y., Tanaka H. (2009). Quantitative Analysis of Ginsenosides Rb1, Rg1, and Re in American Ginseng Berry and Flower Samples by Elisa Using Monoclonal Antibodies. J. Nat. Med..

[64] Fujii S., Tuvshintogtokh I., Mandakh B., Munkhjargal B., Uto T., Morinaga O., Shoyama Y. (2014). Screening of Glycyrrhiza Uralensis Fisch. Ex Dc. Containing High Concentrations of Glycyrrhizin by Eastern Blotting and Enzyme-Linked Immunosorbent Assay Using Anti-Glycyrrhizin Monoclonal Antibody for Selective Breeding of Licorice. J. Nat. Med..

[65] Huihua Q., Feng W., Wenchao S., Xueqian W., Jinjun C., Hui K., Yan Z., Qingguo W. (2015). Pharmacokinetic Analysis of Orally Administered Puerarin in Human Saliva Using an Indirect Competition Elisa. Anal. Methods.

[66] Yeo S.J., Huong D.T., Han J.H., Kim J.Y., Lee W.J., Shin H.J., Han E.T., Park H. (2014). Performance of Coumarin-Derived Dendrimer-Based Fluorescence-Linked Immunosorbent Assay (FLISA) to Detect Malaria Antigen. Malar. J..

[67] Sakamoto S., Taura F., Pongkitwitoon B., Putalun W., Tsuchihashi R., Kinjo J., Tanaka H., Morimoto S. (2010). Development of Sensitivity-Improved Fluorescence-Linked Immunosorbent Assay Using a Fluorescent Single-Domain Antibody against the Bioactive Naphthoquinone, Plumbagin. Anal. Bioanal. Chem..

[68] Zhao Y., Qu H., Wang X., Zhang Y., Cheng J., Zhao Y., Wang Q. (2015). Development of Fluorescence-Linked Immunosorbent Assay for Paeoniflorin. J. Fluoresc..

[69] Shan W., Cheng J., Qu B., Sai J., Kong H., Qu H., Zhao Y., Wang Q. (2015). Development of a Fluorescence-Linked Immunosorbent Assay for Baicalin. J. Fluoresc..

[70] Sakamoto S., Tanizaki Y., Pongkitwitoon B., Tanaka H., Morimoto S. (2011). A Chimera of Green Fluorescent Protein with Single Chain Variable Fragment Antibody against Ginsenosides for Fluorescence-Linked Immunosorbent Assay. Protein Expr. Purif..

[71] Tanaka H., Putalun W., Tsuzaki C., Shoyama Y. (1997). A Simple Determination of Steroidal Alkaloid Glycosides by Thin-Layer Chromatography Immunostaining Using Monoclonal Antibody against Solamargine. Febs Lett..

[72] Shan S., Tanaka H., Shoyama Y. (2001). Enzyme-Linked Immunosorbent Assay for Glycyrrhizin Using Anti-Glycyrrhizin Monoclonal Antibody and an Eastern Blotting Technique for Glucuronides of Glycyrrhetic Acid. Anal. Chem..

[73] Taylor S.C., Posch A. (2014). The Design of a Quantitative Western Blot Experiment. BioMed Res. Int..

[74] Shan S., Tanaka H., Shoyama Y. (1999). Western Blotting Method for the Immunostaining Detection of Glucuronides of Glycyrrhetic Acid Using Anti-Glycyrrhizin Monoclonal Antibody. Biol. Pharm. Bull..

[75] Fukuda N., Tanaka H., Shoyama Y. (2000). Applications of Elisa, Western Blotting and Immunoaffinity Concentration for Survey of Ginsenosides in Crude Drugs of Panax Species and Traditional Chinese Herbal Medicines. Analyst.

[76] Fukuda N., Tanaka H., Shoyama Y. (2001). Double Staining of Ginsenosides by Western Blotting Using Anti-Ginsenoside Rb1 and Rg1 Monoclonal Antibodies. Biol. Pharm. Bull..

[77] Tanaka H., Fukuda N., Shoyama Y. (2006). Identification and Differentiation of Panax Species Using Elisa, Rapd and Eastern Blotting. Phytochem. Anal. PCA.

[78] Morinaga O., Uto T., Sakamoto S., Putalun W., Lhieochaiphant S., Tanaka H., Shoyama Y. (2009). Development of Eastern Blotting Technique for Sennoside A and Sennoside B Using Anti-Sennoside A and Anti-Sennoside B Monoclonal Antibodies. Phytochem. Anal. PCA.

[79] Kajita H. (2007). Practical Use and Technical Notes on Ultrafiltration Membrane for Analysis of Pesticide Residues and Veterinary Drugs in Foods. J. Food Hyg. Soc. Jpn..

[80] Krieter D.H., Lemke H.D. (2011). Polyethersulfone as a High-Performance Membrane. Contrib. Nephrol..

[81] Gomes S.A., Nogueira J.M., Rebelo M.J. (2004). An Amperometric Biosensor for Polyphenolic Compounds in Red Wine. Biosens. Bioelectron..

[82] Putalun W., Fukuda N., Tanaka H., Shoyama Y. (2004). A One-Step Immunochromatographic Assay for Detecting Ginsenosides Rb1 and Rg1. Anal. Bioanal. Chem..

[83] Morinaga O., Fujino A., Tanaka H., Shoyama Y. (2005). An On-Membrane Quantitative Analysis System for Glycyrrhizin in Licorice Roots and Traditional Chinese Medicines. Anal. Bioanal. Chem..

[84] Morinaga O., Fukuda N., Tanaka H., Shoyama Y. (2005). Chromatographic Resolution of Glucosidic Compounds, Ginsenosides on Polyethersulphone Membrane, and Its Application to the Quantitative Immunoassay for Ginseng Saponins. Glycobiology.

[85] Morinaga O., Lu Z., Lin L., Uto T., Sangmalee S., Putalun W., Tanaka H., Shoyama Y. (2013). Detection of Paeoniflorin and Albiflorin by Immunostaining Technique Using Anti-Paeoniflorin Monoclonal Antibody. Phytochem. Anal. PCA.

[86] Morinaga O., Zhu S., Tanaka H., Shoyama Y. (2006). Visual Detection of Saikosaponins by on-Membrane Immunoassay and Estimation of Traditional Chinese Medicines Containing Bupleuri Radix. Biochem. Biophys. Res. Commun..

[87] Fujii S., Morinaga O., Uto T., Nomura S., Shoyama Y. (2016). Development of Double Eastern Blotting for Major Licorice Components, Glycyrrhizin and Liquiritin for Chemical Quality Control of Licorice Using Anti-Glycyrrhizin and Anti-Liquiritin Monoclonal Antibodies. J. Agric. Food Chem..

[88] Ekins R. (1980). More Sensitive Immunoassays. Nature.

[89] Qu H., Wang X., Qu B., Kong H., Zhang Y., Shan W., Cheng J., Wang Q., Zhao Y. (2016). Sandwich Enzyme-Linked Immunosorbent Assay for Naringin. Anal. Chim. Acta.

[90] Putalun W., Tanaka H., Shoyama Y. (2005). Rapid Detection of Glycyrrhizin by Immunochromatographic Assay. Phytochem. Anal. PCA.

[91] Qu H., Zhang Y., Qu B., Kong H., Qin G., Liu S., Cheng J., Wang Q., Zhao Y. (2016). Rapid Lateral-Flow Immunoassay for the Quantum Dot-Based Detection of Puerarin. Biosens. Bioelectron..

[92] Paudel M.K., Putalun W., Sritularak B., Morinaga O., Shoyama Y., Tanaka H., Morimoto S. (2011). Development of a Combined Technique Using a Rapid One-Step Immunochromatographic Assay and Indirect Competitive Elisa for the Rapid Detection of Baicalin. Anal. Chim. Acta.

[93] Putalun W., Morinaga O., Tanaka H., Shoyama Y. (2004). Development of a One-Step Immunochromatographic Strip Test for the Detection of Sennosides A And B. Phytochem. Anal. PCA.

[94] Zhang Y., Xiao J., Wang Q., Zhang Y. (2016). A Modified Quantum Dot-Based Dot Blotting Assay for Rapid Detection of Fish Pathogen Vibrio Anguillarum. J. Microbiol. Biotechnol..

[95] Kurien B.T., Scofield R.H. (2015). Other Notable Methods of Membrane Protein Detection: A Brief Review. Methods Mol. Biol..

[96] Li B., Yu Q., Duan Y. (2015). Fluorescent Labels in Biosensors for Pathogen Detection. Crit. Rev. Biotechnol..

[97] Le T., Xie Y., Zhu L., Zhang L. (2016). Rapid and Sensitive Detection of 3-Amino-2-Oxazolidinone Using a Quantum Dot-Based Immunochromatographic Fluorescent Biosensor. J. Agric. Food Chem..

[98] Zhao Y., Feng H., Shan W., Cheng J., Wang X., Zhao Y., Qu H., Wang Q. (2015). Development of Immunoaffinity Chromatography to Specifically Knockout Baicalin from Gegenqinlian Decoction. J. Sep. Sci..

[99] Jiang J.G. (2009). Identification and Quality Control of Chinese Medicine Based on the Fingerprint Techniques. Curr. Med. Chem..

[100] Yanagihara H., Sakata R., Minami H., Tanaka H., Shoyama Y., Murakami H. (1996). Immunoaffinity Column Chromatography against Forskolin Using an Anti-Forskolin Monoclonal Antibody and Its Application. Anal. Chim. Acta.

[101] Putalun W., Tanaka H., Shoyama Y. (1999). Rapid Separation of Solasodine Glycosides by an Immunoaffinity Column Using Anti-Solamargine Monoclonal Antibody. Cytotechnology.

[102] Yuan C.S., Tanaka H. (2011). Bioactivity of American Ginseng by Knockout Extract Preparation Using Monoclonal Antibody. Curr. Drug Discov. Technol..

[103] Uto T., Morinaga O., Tanaka H., Shoyama Y. (2012). Analysis of the Synergistic Effect of Glycyrrhizin and Other Constituents in Licorice Extract on Lipopolysaccharide-Induced Nitric Oxide Production Using Knock-Out Extract. Biochem. Biophys. Res. Commun..

[104] He T., Wang Y., Li P., Zhang Q., Lei J., Zhang Z., Ding X., Zhou H., Zhang W. (2014). Nanobody-Based Enzyme Immunoassay for Aflatoxin in Agro-Products with High Tolerance to Cosolvent Methanol. Anal. Chem..

[105] Ma L., Sun Y., Kang X., Wan Y. (2014). Development of Nanobody-Based Flow Injection Chemiluminescence Immunoassay for Sensitive Detection of Human Prealbumin. Biosens. Bioelectron..

